# Confidence intervals and tests are two sides of the same research question

**DOI:** 10.3389/fpsyg.2015.00034

**Published:** 2015-01-28

**Authors:** Jose D. Perezgonzalez

**Affiliations:** Business School, Massey UniversityPalmerston North, New Zealand

**Keywords:** confidence interval, CI, significance testing, NHST, Fisher, Neyman-Pearson

Cumming's (2014) article was commissioned by *Psychological Science* to reinforce the journal's 2014 publication guidelines. It exhorts substituting confidence intervals (CIs) for Null Hypothesis Significance Testing (NHST) as a way of increasing the scientific value of psychological research. Cumming's article is somehow biased, hence the aims of my commentary: to balance out the presentation of statistical tests and to fend CIs against misinterpretations. Researchers with an interest in the correct philosophical application of tests and CIs are my target audience.

## Technical tests

NHST is a philosophical mismatch of three incompatible theories: Fisher's, Neyman-Pearson's, and Bayes's (Gigerenzer, 2004). Technologically, however, it reduces to either of the former two, most often to Fisher's (Cortina and Dunlap, 1997). Few researchers concern themselves with the philosophical underpinnings of NHST and rather use it as a mere technology. Therefore, it is more interesting to discuss Fisher's (1954) or Neyman and Pearson's theories (1933) than NHST. Cumming does so in his book (2012) but not in his article, thus painting an inaccurate picture of the value of data testing in research.

In a nutshell, Fisher's relevant constructs are null hypotheses, levels of significance and *ad hoc p*-values. Neyman-Pearson's relevant constructs are main and alternative hypotheses, long-run errors under repeated sampling (α, β), critical test values, and power (1–β)—*p*-values are not relevant here but can be used as proxies for deciding between hypotheses (Perezgonzalez, 2014).

Cumming's Figure 1 about the dance of p's (alternatively, http://youtu.be/ez4DgdurRPg) is not suitable for representing Fisher's *ad hoc* approach (and, by extension, most NHST projects). It is, however, adequate for representing Neyman-Pearson's repeated sampling approach, a simple count of significant tests irrespective of their *p*-value. As it turns out, Cumming's figure is a textbook example of what to expect given power (Table 1). For example, 48% of tests are significant at α = 0.05 out of 50% expected.

**Table 1:** **Expected and observed significant tests given α and 1–β**.

**α**	**Expected (1–β)**	**N sig tests**	**Observed**
0.05	0.50	12	0.48
0.01	0.26	7	0.28
0.10	0.63	1	0.68
0.20	0.76	19	0.76

Based on Cumming's (2014), data; calculated with G * Power.

## Fair comparison

Cumming writes “CIs and *p* values are based on the same statistical theory” (p. 13), which is partly correct. CIs were developed by Neyman (1935) and, thus, CIs and Neyman-Pearson's tests are grounded on the same statistical philosophy: repeated sampling from the same population, errors in the long run (1−CI = β), and assumption of true population parameters (which are unknown in the case of CIs)—*p*-values, however, are part of Fisher's theory.

CIs and Neyman-Pearson's tests are, thus, two sides of the same coin. Tests work on the main hypotheses, with known population parameters but unknown sample probabilities, and calculate point estimates to make decisions about those samples. CIs work on the alternative hypotheses, with known sample interval probabilities but unknown population parameters, and calculate CIs to describe those populations.

To be fair, a comparison of both requires equal ground. At interval level, CIs compare with power, and Cumming's figure reveals that 92% of sample statistics fall within the population CIs (95% are expected) versus the power results presented in Table 1. At point estimate level, means (or a CI bound) compare with *p*-values, and Cumming's figure reveals a well-choreographed dance between those. Namely, CIs are not superior to Neyman-Pearson's tests when properly compared although, as Cumming discussed, CIs are certainly more informative.

## Fending against fallacies

The common philosophy underlying CIs and tests implies that they share similar fallacies. Cumming touches on some but does not pre-emptily resolve them for CIs.

Cumming writes, “if *p* reveals truth… ” (p. 12). It is not clear what truth Cumming refers to, most probably about two known fallacies: that *p* is the probability of the main hypothesis being true and, consequently, that 1–*p* is the probability of the alternative hypothesis being true (e.g., Kline, 2004). Similar fallacies equally extend to the power of the alternative hypothesis. Yet accepting the alternative hypothesis does not mean a 1–β probability of it being true, but a probability of capturing 1–β samples pertaining to its population in the long run. The same can be said about CIs (insofar CI = 1–β): they tell something about the data—about their probability of capturing a population parameter in the long run—not about the population—i.e., the observed CI may not actually capture the true parameter.

Another fallacy touched upon is that *p* informs about replication. *P*-values only inform about the *ad hoc* probability of data under the tested hypothesis, thus “(they) have little to do with replication in the usual scientific sense” (Kline, 2004, p. 66). Similarly, CIs do not inform about replicability either. They are a statement of expected frequency under repeated sampling from the same population. The 83% next-experiment replicability reported by Cumming (2014), although interesting, is not part of the frequentist understanding of CIs and seems explainable by the size of the confidence interval.

## Fending against mindless use of CIs

Finally, there is the ever present risk that CIs will be used as mindlessly as tests. For one, CIs share the same philosophical background than Neyman-Pearson's tests, yet many researchers take them to mean an *ad hoc* probability of personal confidence (Hoekstra et al., 2014). On the other hand, the inferential value of a CI rests on the assumption that the unknown population parameter has equal chance of being anywhere within that interval. Using a point estimate leads to the wrong conclusion: that such point estimate is more probable than any other in the interval.

## Final note

Both CIs and tests are useful tools (Gigerenzer, 2004). Neyman's CIs are more descriptive, Fisher's tests find significant results and foster meta-analyses, Neyman-Pearson's tests help with decisions, NHST means trouble. Yet, to keep Psychology at the frontier of science we ought to consider alternative tools in the statistical toolbox, such as exploratory data analysis (Tukey, 1977), effect sizes (Cohen, 1988), meta-analysis (Rosenthal, 1984), cumulative meta-analysis (Braver et al., 2014), and Bayesian applications (Dyjas et al., 2012; Barendse et al., 2014), lest we forget.

### Conflict of interest statement

The author declares that the research was conducted in the absence of any commercial or financial relationships that could be construed as a potential conflict of interest.
